# What Darwin could not see: island formation and historical sea levels shape genetic divergence and island biogeography in a coastal marine species

**DOI:** 10.1038/s41437-023-00635-4

**Published:** 2023-07-03

**Authors:** Maximilian Hirschfeld, Adam Barnett, Marcus Sheaves, Christine Dudgeon

**Affiliations:** 1 College of Science and Engineering, James Cook University, Townsville, Queensland Australia; 2 Galápagos Science Center, Universidad San Francisco de Quito, Isla San Cristóbal, Galápagos Ecuador; 3 Marine Data Technology Hub, James Cook University, Townsville, Queensland Australia; 4 Biopixel Oceans Foundation, Cairns, Queensland Australia; 5 School of Biomedical Sciences, The University of Queensland, Saint Lucia, Queensland Australia

**Keywords:** Structural variation, Conservation genomics, Biogeography, Molecular ecology, Evolutionary ecology

## Abstract

Oceanic islands play a central role in the study of evolution and island biogeography. The Galapagos Islands are one of the most studied oceanic archipelagos but research has almost exclusively focused on terrestrial organisms compared to marine species. Here we used the Galapagos bullhead shark (*Heterodontus quoyi*) and single nucleotide polymorphisms (SNPs) to examine evolutionary processes and their consequences for genetic divergence and island biogeography in a shallow-water marine species without larval dispersal. The sequential separation of individual islands from a central island cluster gradually established different ocean depths between islands that pose barriers to dispersal in *H. quoyi*. Isolation by resistance analysis suggested that ocean bathymetry and historical sea level fluctuations modified genetic connectivity. These processes resulted in at least three genetic clusters that exhibit low genetic diversity and effective population sizes that scale with island size and the level of geographic isolation. Our results exemplify that island formation and climatic cycles shape genetic divergence and biogeography of coastal marine organisms with limited dispersal comparable to terrestrial taxa. Because similar scenarios exist in oceanic islands around the globe our research provides a new perspective on marine evolution and biogeography with implications for the conservation of island biodiversity.

## Introduction

Oceanic archipelagos are ideal model systems to study evolutionary processes and their consequences on genetic variation in the light of island formation (Emerson 2002; Parent et al. 2008; Warren et al. 2015). Individual islands of archipelagos constitute independent experimental units to examine how barriers to dispersal arrange the spatial genetic variation in natural populations (Emerson 2002; Parent et al. 2008). Two principal evolutionary mechanisms can be observed in oceanic archipelagos. The first is rapid adaptation, which leads to genetic differentiation through selection and the exploitation of diverse ecological niches after colonization of new island environments, often in the absence of obvious physical barriers (Schluter and Conte 2009; Langerhans and Riesch 2013). In the second instance, divergence is generated through genetic drift among populations that are separated by physical barriers to dispersal (Avise 2000). Barriers can be established through vicariance events dividing previously connected populations or founder populations separate through the colonization of new habitat by dispersing across existing barriers (Sanmartín 2003; Cowie and Holland 2006). The geographic isolation through barriers results in distinct genetic signatures that are exacerbated in island populations (Frankham 1998). The low standing genetic variation of few founding individuals and limited dispersal between small fragmented patches of habitat that provide limited resources result in low genetic diversity and small population sizes (Frankham 1996, 1997; Brüniche-Olsen et al. 2019).

In volcanic archipelagos individual islands are formed sequentially through recurring volcanic activity as tectonic plates move across hotspots. The progression rule describes the sequential colonization and subsequent genetic divergence from older towards younger volcanic islands in terrestrial organisms (Fleischer et al. 1998; Shaw and Gillespie 2016). However, a progressive divergence may be absent in island species that have high levels of inter-island dispersal, arrived recently or through multiple colonization events, or underwent strong divergent selection (Juan et al. 2000; Parent et al. 2008; Shaw and Gillespie 2016). Strong sea level fluctuations of the late Quaternary period altered island configuration at faster rates and shorter geological time scales than plate movement, but the impact of this process on divergence and island biogeography remains largely unclear (Fernández-Palacios et al. 2016; Weigelt et al. 2016). Island biogeography of marine organisms differs from their terrestrial counterparts because higher dispersal in the marine environment increases inter-island connectivity and rates of immigration to oceanic archipelagos (Pinheiro et al. 2017). This can result in faster occupation of ecological niches, leaving fewer opportunities for adaptive radiation and in-situ divergence (Pinheiro et al. 2017).

In the ocean, just as on land, dispersal regulates population connectivity and is therefore a fundamental driver of biogeographic and genetic patterns in oceanic islands (Cowie and Holland 2006). But what constitutes a physical barrier to dispersal in the ocean is largely dependent on the mode of dispersal (Hachich et al. 2015; Hirschfeld et al. 2021). A diverse array of marine barriers, including mid ocean barriers, current fronts, and strong salinity or temperature gradients, have been shown to restrict gene flow in marine organisms with planktonic larvae (Rocha et al. 2007). In contrast, marine taxa that lack planktonic larvae, including mammals, reptiles and elasmobranchs (sharks, skates and rays), depend on active migration of individuals to maintain genetic connectivity (Dudgeon et al. 2012; Hirschfeld et al. 2021). Many pelagic sharks undertake large-scale transoceanic and inter-oceanic migrations (Block et al. 2011) and oceanic and deep sea species can maintain genetic connectivity between continental coasts and oceanic islands (Gubili et al. 2016; Domingues et al. 2018), and across major ocean basins (Catarino et al. 2015; Veríssimo et al. 2017). Ocean depth between shallow coastal habitat, however, is a strong barrier to dispersal in some shallow-water benthic sharks and rays, generating genetic differences between individual islands at extremely small spatial scales (Gaida 1997; Plank et al. 2010). Shallow-water marine organisms that lack dispersive larvae are therefore likely to produce unique genetic and biogeographic patterns in oceanic archipelagoes compared to terrestrial organisms and marine species with planktonic dispersal, but have rarely been studied (Cowie and Holland 2006).

**Fig. 1:**
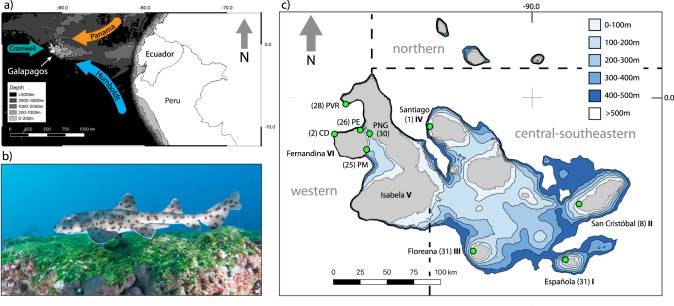
Overview of the study system. **a** Location of the Galapagos Islands in the Eastern Tropical Pacific in relation to ocean bathymetry, the Panama, Humboldt, and Cromwell Currents; **b** Adult male Galapagos bullhead shark (*Heterodontus quoyi*); **c** Sampling design: Sampling locations (green circles) with number of samples in brackets. Sequential island emergence is given in bold roman letters next to island names. Dashed lines and gray letters indicate the separation between the Western, Central-Southeastern and northern bioregions. PVR Punta Vicente Roca, PNG Parque Nacional Galapagos, PE Punta Espinoza, PM Punta Mangle, CD Cabo Douglas.

The Galapagos archipelago and the Galapagos bullhead shark (*Heterodontus quoyi*) provide a unique model system for the study of evolutionary processes in oceanic islands. The volcanic islands are separated from the South American coast by approximately 1000 km of up to 2000 m deep ocean (Fig. 1a). The eastward movement of the Nasca plate across the Galapagos hotspot resulted in the sequential formation of volcanic islands and the complex bathymetry of the Galapagos plateau (Snell et al. 1996; Brewington et al. 2014). The convergence of three major ocean currents creates contrasting oceanographic conditions and diverse marine biogeography on a small spatial scale (Edgar et al. 2004). In the Galapagos, terrestrial organisms with limited inter-island dispersal generally follow a progressive genetic divergence that reflects the sequential formation of clusters of islands with similar age (Parent et al. 2008). Recent paleogeographic reconstructions of the Galapagos account for the periodical fusion and fission of landmasses through historical sea-level fluctuations, which has left its footprint on the biogeography of terrestrial organisms (Ali and Aitchison 2014; Karnauskas et al. 2017). Here we use a shallow-water benthic shark to test if paleogeographic dynamics also influence genetic divergence in coastal marine organisms with limited dispersal. The Galapagos bullhead shark (Fig. 1b) has a small geographic range comprising the central-southeastern and western Galapagos archipelago and the continental shelf of northern Perú and possibly southern Ecuador (Bearez 1996; Acuña-Marrero et al. 2018). The species is thought to be absent from the northern and far-northern bioregions that are separated from the main Galapagos platform by water over 1000 m deep and are characterized by warmer water of the Panama Current (Edgar et al. 2004; Acuña-Marrero et al. 2018). Currently, no primary scientific literature exists on the biology and ecology of *H. quoyi*. All species in the genus Heterodontus are oviparous and the Galapagos bullhead shark likely has a long generation time, slow growth rates and late age at maturity similar to other Heterodont sharks (Powter and Gladstone 2008). For example, Port Jackson sharks (*Heterodontus portusjacksoni*) have a generation time of 22.5 years, slow adult growth rates (31.4– 32.7 mm year^−1^) and females mature between 8 and 10 and males between 11 and 14 years (McLaughlin and O’Gower 1971; Powter and Gladstone 2008)*.* The small-bodied shark (<100 cm) has a strictly benthic lifestyle, spending the majority of time on or close to the ocean floor often hiding in caves and crevices, particularly juveniles, and inhabiting rocky reefs in proximity to cold-water upwelling areas at less than 40 m depth (Weigmann 2016; Acuña-Marrero et al. 2018). Here we test the hypothesis that ocean depth is a barrier to dispersal and gene flow in Galapagos bullhead sharks and assess the role of island formation and historical sea level changes in shaping genomic signatures of geographic isolation.

## Materials and methods

### Study area and sampling design

To examine the role of ocean bathymetry, island formation and historical sea level fluctuations on spatial genetic structure, Galapagos bullhead sharks were sampled from six islands in the Galapagos archipelago (Fig. 1) that are separated by varying levels of depth and emerged at different geological times, in an approximate East to West sequence (Geist et al. 2014; Karnauskas et al. 2017). We also sampled multiple locations on the same island (Fernandina and Isabela) that are connected by continuous shallow-water habitat to evaluate potential genetic isolation by distance (IBD) due to limited dispersal capacity in absence of depth barriers. Sampling locations located within 5 km of each other, were combined. Sharks were captured and released by hand during SCUBA to collect tissue samples from fin clips. Underwater sampling was designed to minimize handling time and stress responses in the sharks (see *Ethics Statement*). A total of 33 locations on seven islands were visited between 2015 and 2018 (Supplementary Information Fig. S1) to search for *H. quoyi*. Locations that revealed higher abundances of sharks were visited repeatedly to increase the number of samples per location and achieve a balanced sampling design.

### SNP genotyping and quality control (see Supplementary Information)

DNA extraction, sequencing and SNP genotyping was conducted by Diversity Array Technologies (DArT, Canberra, Australia). Samples were processed using the proprietary DArT Pty Ltd analytical pipeline, which includes technical replicates from a subset of samples to assess genotyping reproducibility (Georges et al. 2018). The following quality control steps were applied in addition to the DArT pipeline to avert potential downstream effects of SNP selection on the inference of population structure in non-model organisms (O’Leary et al. 2018). We randomized tissue samples from all sampling locations across sequencing plates and replicated samples from two individuals within and across plates to independently assure genotyping consistency and generate baseline values to quickly assess relatedness and potential sample contamination during data filtering (Meirmans 2015; O’Leary et al. 2018). To assure high quality of our SNP data set for reliably and adequately assessing population structure in relation to our hypotheses we filtered the raw data set using the R package *radiator* (Gosselin 2019). Markers that are putatively under selection (outlier SNPs) were identified using two approaches suited for spatially structured populations, PCadapt (Luu et al. 2017) and OutFLANK (Whitlock and Lotterhos 2015). Loci identified as putative outliers with both methods were then removed to create a neutral SNP data set to assess genetic patterns generated through neutral processes.

### Population structure

Population structure was assessed using Bayesian clustering and pairwise fixation and differentiation indices. First, neutral SNPs were analyzed to infer proportions of genetic admixture for all individuals from *K* hypothetical ancestral populations using the R package *tess3r* (Caye et al. 2016). This method uses spatially explicit Bayesian models and is free from assumptions about minor allele frequency, Hardy–Weinberg equilibrium and linkage disequilibrium. Individual admixture proportions were estimated for *K* ancestral populations between one and eight, with ten replicate runs for each value of *K*, 10,000 iterations, a tolerance value of 10^−6^ and a spatial parameter, alpha, equals 0.01. As alpha approximates zero the algorithm produces results approximating those of the program STRUCTURE (Caye et al. 2016). The most likely number for *K* ancestral populations was estimated based on the lowest value of the cross-entropy criterion (Frichot et al. 2014), generated using the cross-validation method and masking 10% of the data in training data sets (Caye et al. 2016). Because unequal sample sizes in our data set may bias admixture results we repeated the analysis, with the same parameters, on a subsample of 56 individuals. For locations with more than eight samples a subset of individuals were randomly chosen and all samples were kept for all locations with less than eight samples. The data set contained ten individuals from Española, Floreana, and Punta Vicente Roca (Isabela Island), five from Punta Espinoza (Fernandina Island), Punta Mangle (Fernandina Island) and Parque Nacional Galapagos (Isabela Island), eight from San Cristóbal, two from Cabo Douglas (Fernandina Island) and one from Santiago Island. We calculated pairwise fixation, *F*_ST_ (Weir and Cockerham 1984), and differentiation, *D*_ST_ (Jost 2008), indices between all locations with at least eight samples for neutral SNPs using the R package *strataG* (Archer et al. 2017). Significance of pairwise comparisons and corresponding p-values were calculated based on 1000 bootstraps and corrected for false positives using the FDR correction (Benjamini and Hochberg 1995) implemented in the *p.adjust* R base function (R Core Team 2019). We tested for directional gene flow among sampling locations with at least eight samples using relative migration rates (based on *Nei’s G*_*ST*_ and 1000 bootstraps) calculated for neutral SNPs with the *divMigrate* function of the *diveRsity* R package (Keenan et al. 2013; Sundqvist et al. 2016). Finally, we examined the relationship between spatial genetic patterns and progressive island formation using the most recent information on approximate age of emergence (Geist et al. 2014) and paleogeographic reconstructions of the Galapagos (Ali and Aitchison 2014; Karnauskas et al. 2017).

### Isolation by depth

To test for the effect of contemporary bathymetry and historical sea level fluctuations on genetic connectivity we adapted isolation by resistance (IBR) analysis (McRae 2006) using depth profiles of the Galapagos to represent landscape resistance to animal dispersal (see Supplementary Information). Briefly, two IBR models were built using single surface optimization in the *ResistanceGA* R package, one based on contemporary bathymetry and another based on paleogeographic bathymetry that accounts for historical sea level fluctuations (Peterman 2018). We compared the resistance models to a null model based on isolation by distance analyses that only uses geographic distance and does not account for potential depth barriers (Slatkin 1993; Guillot et al. 2009). Paleogeographic models of the Galapagos archipelago spanning the last 700,000 years show that sea levels were repeatedly between 145 and 210 m deeper during glacial maxima (Ali and Aitchison 2014). To test if low sea levels during glacial periods may have facilitated historical dispersal, the paleogeographic IBR model was built by optimizing a bathymetry layer where all areas between 0 and −210 m, corresponding to areas with shallow water depth at least one time during the last 700,000 years, were assigned a shallow water depth of 1 m. Resistance distances (Shah and McRae 2008) were calculated for optimized contemporary and paleogeographic resistance surfaces with the *ResistanceGA* package (Peterman 2018). *ResistanceGA* uses a genetic algorithm to optimize resistance surfaces based on pairwise genetic data and resistance distances generated through Circuitscape software (Peterman 2018; Anantharaman et al. 2019). The linear relationship between linearized genetic distances (FST/(1−FST) and straightest over-water distances and between linearized genetic distances and contemporary and paleogeographic resistance distances was plotted and quantified using Pearson’s correlation coefficient (*r*^2^) and Mantel tests with 1000 permutations with the R package *DartR* (Gruber and Georges 2019). The performance of each model was compared using a causal modeling approach (McRae and Beier 2007).

### Signatures of isolation

To assess signatures of isolation we calculated several genetic diversity indices for sampling locations with at least eight samples using the neutral SNP data set and the *diveRsity* R package (Keenan et al. 2013). Indices included allelic richness (*A*_*R*_), observed heterozygosity (*H*_O_), expected heterozygosity (*H*_*E*_), and inbreeding coefficients (*F*_*IS*_). We estimated local contemporary genetic effective population size (*N*_*e*_) for each genetically distinct unit with more than eight individuals identified by clustering analyses using the bias-corrected linkage disequilibrium (LD) method implemented in NeEstimator v.2.01 (Waples and Do 2008; Do et al. 2014). The method assumes closed populations, no mutation or selection and can provide robust estimates of local *N*_*e*_ if migration rates between demes are low (Waples and Do 2008, 2010). The likelihood that SNP loci are physically linked, potentially biasing *N*_*e*_ estimates based on LD, is low in our data set because we only kept one SNP locus per sequence and because of the large genome size in heterodont sharks (Akey et al. 2001; Waples 2006; Waples et al. 2016). Sharks in each cluster also covered a large range of size classes, spanning several generations, which reduces downward bias in single sample *N*_*e*_ estimates based on LD in organisms with overlapping generations (Waples et al. 2014). We determined the best minor allele frequency threshold (*Pcrit*) for each individual genetic cluster using the formula 1/(2S) < *Pcrit* < = 1/S (S=number of samples) as suggested by (Waples and Do 2010). Because no life history parameters are known for *H. quoyi* no correction based on life span and maturity were be applied. Finally, we corrected *N*_*e*_ estimates for the number of haploid chromosomes found in the congeneric *H. japonicus* and *H. francisci (n* = *51)* using the formula *N*_*ecor*_ = *N*_*e*_*/(0.098* + *0.219* × *lnChr)*, where *Chr* is the number of chromosomes (Stingo and Rocco 2001; Waples et al. 2016).

## Results

### Sampling and genotyping

A total of 182 sharks were sampled from nine locations on six islands. Between 24 and 30 individuals were collected at each of four locations in the western bioregion (Fernandina and Isabela Islands) and on each of two islands in the central-southeastern bioregion (Española and Floreana Islands). Despite exploring eight locations on 20 dives between 2015 and 2018 only eight individuals were sampled on San Cristóbal Island. Only a single individual was captured after diving at six locations around Santiago Island, which was not revisited after. The DArT pipeline generated 33606 SNPs and after quality filtering we retained a total of 9223 neutral SNPs and 180 individuals (Supplementary Information Table S1). Briefly, none of the samples had large amounts of missing data and none showed signs of cross contamination or sample degradation based on excessively low or high heterozygosity. Two sharks, one on Fernandina and another on Isabela Island, were recaptured at the same location after a period of approximately one year. The individuals were identified as duplicate samples during the filtering process. For each pair of resampled sharks we kept the sample with less missing data (higher call rate). We removed five putatively female-linked SNPs that had higher heterozygosity in females. These SNPs were present in around 50% of males compared to females indicating male heterogamety in Galapagos Bullhead sharks. Eleven loci putatively under selection were recovered by both outlier methods (75 SNPs in PCadapt, 14 SNPs in OutFLANK).

### Population structure

**Fig. 2:**
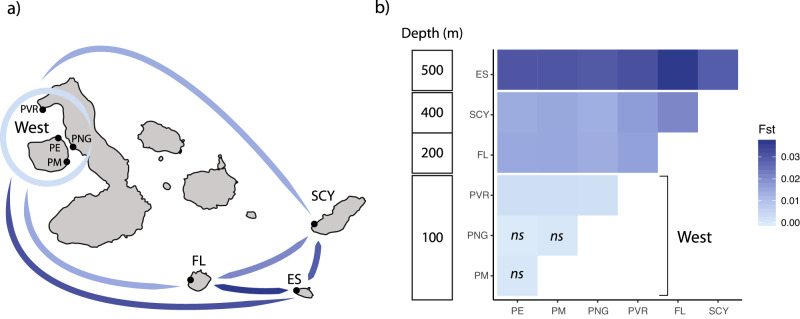
Pairwise comparisons (*F*_*ST*_) between sampling locations based on 9223 neutral SNPs. **a** Visualization of the level of genetic differentiation between locations. Comparisons between the western locations and other islands and inter-island comparisons are indicated by blue lines with color intensity increasing with *F*_*ST*_ values. Low levels of genetic differentiation among the western locations are indicated by a light blue circle. **b** Minimum ocean depth (vertical bar) and levels of *F*_*ST*_ between locations (ES Española, SCY San Cristóbal, FL Floreana, PVR Punta Vicente Roca, PNG Parque National Galapagos, PM Punta Mangle, PE Punta Espinoza).

Pairwise comparisons using neutral SNPs showed increasing genetic differentiation with greater depth among sampling locations and older island emergence time (Fig. 2; Supplementary Information Table S4). Española Island, the oldest and most isolated island, consistently exhibited the highest genetic differentiation from all other locations. San Cristóbal and Floreana Islands showed the second highest differentiation. San Cristóbal Island revealed similar levels of differentiation (*F*_ST_: 0.0133–0.0169) with the western locations compared to Floreana Island (*F*_ST_: 0.0139–0.0161) based on *F*_ST_ values, but higher differentiation using *D*_ST_ (0.00038–0.00047 vs. 0.0003–0.00034). All pairwise comparisons within the western region were low for *F*_ST_ (0.0003–0.0028) and *D*_ST_ (0.000094–0.00014). With the exception of Punta Vicente Roca, the northernmost location on Isabela Island, all pairwise comparisons in the western region were not significant before and after false discovery rate (FDR) correction. We found no significant directional gene flow among any pair of sampling locations based on relative migration rates of the *divMigrate* method.

**Fig. 3:**
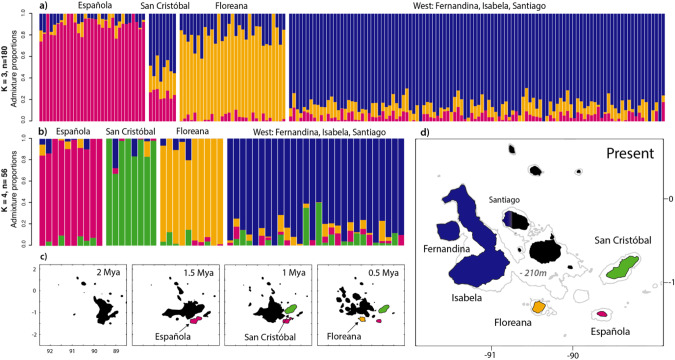
Population structure and island formation. **a** Individual admixture proportions for 180 individuals, 9223 neutral SNPs and *K* = 3 ancestral populations and **b** individual admixture proportions for 56 individuals, 9223 neutral SNPs and *K* = 4 ancestral populations, ordered from left to right following sequential island separation. **c** Hypothetical paleogeographic formation of the Galapagos archipelago with sequential separation of Española Island (purple) at 2–1.5 million years ago (Mya), San Cristóbal Island (green) at 1.5–1 Mya, and Floreana Island (yellow) at 1–0.5 Mya, and the emergence of individual islands that formed Isabela and Fernandina Islands since 0.5 Mya, adapted from (Karnauskas et al. 2017). **d** Present day Galapagos with 210 m isobath indicating the land area that was exposed at the lowest sea level during the last 700,000 years.

Admixture analyses of 9223 neutral SNPs for all 180 sharks identified the most likely number of ancestral populations (*K*) as three (Fig. 3a and Supplementary Information Fig. S4). Sharks from Española and Floreana Islands formed two distinct ancestral populations, and individuals from all locations in the western archipelago (Isabela and Fernandina Islands) together with Santiago Island formed a third ancestral population. Individuals from San Cristóbal showed approximately one third genetic admixture from each of the three ancestral populations. Using the more balanced subset of 56 sharks, the admixture analyses showed that sharks sampled from Española, San Cristobal, Floreana Islands, and the western archipelago with Santiago Island had similar admixture proportions corresponding to four distinct ancestral populations (Fig. 3b; Supplementary Information Fig. S5), although the cross-entropy criterion did not identify any number of ancestral populations to be more likely than *K* = 1. Examination of admixture results in the light of paleogeographic reconstructions displayed that ancestral populations correspond to individual islands that sequentially separated from a central island cluster over the last two million years (Fig. 3c, d). Española Island was differentiated first, at *K* = 2 ancestral populations. Although Floreana Island is differentiated next, at *K* = 3, San Cristóbal Island carries larger amount of admixture from the most ancestral population, Española. Individuals from San Cristóbal Island had similar levels of admixture from Floreana Island, the western archipelago and Española Island at *K* = 3, using the full and balanced data sets, and were assigned a distinct ancestral population at *K* = 4 using the more balanced data set (Fig. 3b).

### Isolation by depth

**Fig. 4:**
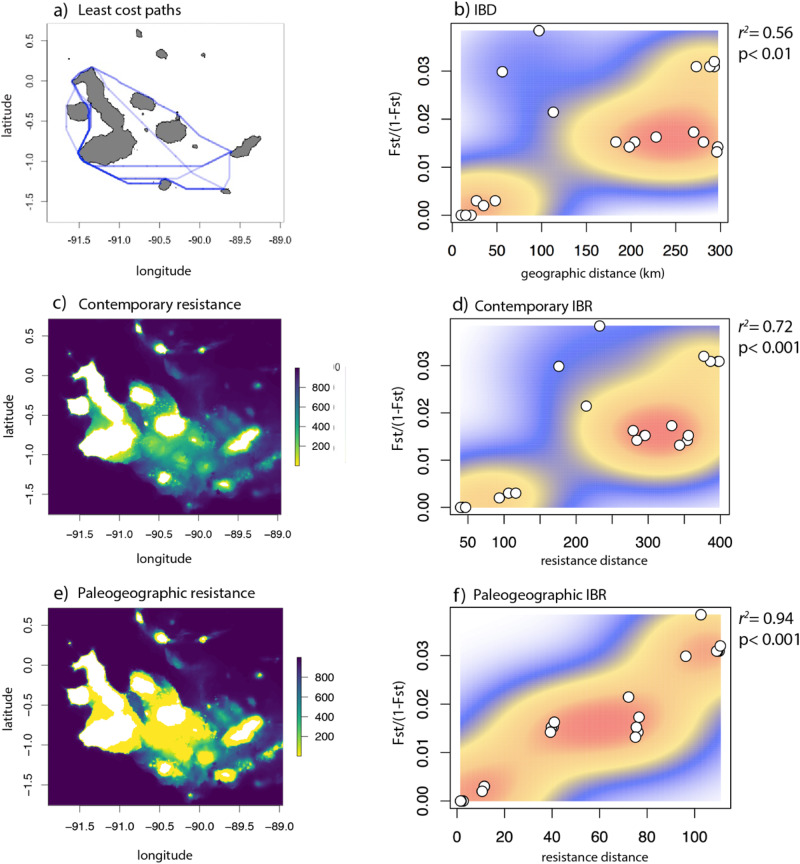
Comparison between IBD (isolation by distance), contemporary IBR (isolation by resistance), and paleogeographic IBR (isolation by resistance) models. **a** Least-cost paths for shortest over-water distance between pairs of locations. **b** Scatter plot with heatmap visualization of the correlation between geographic distance and genetic distance. **c** Optimized resistance surface based on contemporary bathymetry of the Galapagos archipelago. **d** Correlation between contemporary resistance distance and genetic distance. **e** Optimized resistance surface based on paleogeographic bathymetry of the Galapagos archipelago. **f** correlation between paleogeographic resistance distance and genetic distance. Darker colors in resistance surfaces represent higher resistance for the hypothetical movement of sharks among locations and bright yellow shading lower resistance, corresponding to coastal areas that were exposed or had a shallow water depth during glacial maxima.

Model comparison showed that although all three models presented a significant relationship with genetic distance, the model fit improved when considering contemporary bathymetry, and accounting for historical sea level fluctuations provided the best model fit (Fig. 4, Supplementary Information Table S2 and 3). IBD based on shortest over-water distances between sampling locations resulted in significant correlation with genetic distance (mantel rest: *r*^*2*^ = 0.56, *p* < 0.01). However, the scatter plot revealed a large gap between western and southeastern locations, indicating the presence of barriers between those locations and pairwise genetic distances among southeastern locations were high despite their geographical proximity. Single surface optimization using *ResistanceGA* selected and applied reverse monomolecular transformation to the contemporary and paleogeographic bathymetry surfaces (Fig. 4c, e) and generated pairwise resistance distances based on the optimized resistance surfaces (Supplementary Information Figs. S2 and S3). Contemporary IBR, which accounted for ocean depth between sampling locations, resulted in a higher correlation with genetic distance (Mantel test: *r*^*2*^: 0.72, *p* < 0.001), compared to the IBD model. The scatter plot displays that the model partially accounts for depth barriers, providing a better fit for the data. Finally, the paleogeographic IBR model revealed the strongest correlation with genetic distances (Mantel test: *r*^*2*^: 0.94, *p* < 0.001) by taking into account ocean depth and historical sea level oscillations (Fig. 4e, f).

### Genomic signatures of isolation

Genomic diversity was similarly low for all sampling locations. Allelic richness (*A*_R_) ranged from 1.31 to 1.39 and observed heterozygosity (*H*_O_) from 0.091 to 0.098 (Table 1). Similar levels of expected and observed heterozygosity and inbreeding coefficients (*F*_IS_) close to zero indicate the absence of recent bottlenecks.

**Table 1:** Genetic diversity of Galapagos bullhead sharks (*Heterodontus quoyi*) based on 9223 neutral SNPs.

Location	*n*	*A*_R_	*H*_O_	*H*_E_	*F*_IS_
Española (ES)	30	1.37	0.098	0.098	0.004
San Cristobal (SCY)	8	1.31	0.089	0.089	−0.007
Floreana (FL)	29	1.36	0.091	0.093	0.013
Punta Espinoza (PE)	25	1.39	0.097	0.097	0
Punta Mangle (PM)	24	1.38	0.096	0.095	−0.002
Parque Nacional Galapagos (PNG)	30	1.39	0.096	0.096	0
Punta Vicente Roca (PVR)	26	1.39	0.098	0.098	0.003

*n* sample size, *A*_R_ allelic richness, *H*_O_ observed heterozygosity, *H*_E_ expected heterozygosity, *F*_IS_ inbreeding coefficient.

Genetic effective population size (*N*_*e*_) was corrected for a 4% downward bias (*N*_*ecor*_ = *N*_*e*_/0.959) to account for the number of chromosomes based on the congeneric *H. japonicus* and *H. francisci*. This resulted in the lowest *N*_*ecor*_ for Española, the most isolated and smallest island, followed by Floreana islands, which is geographically less isolated and about three times the size (Table 2). The largest effective population size was estimated for combined locations on Isabela and Fernandina islands (West) that formed a single population based on clustering analyses.

**Table 2:** Genetic effective population sizes (*N*_*e*_) with confidence intervals (CI) and corrected effective population sizes (*N*_*ecor*_) for the three main genetic clusters.

Location (sample size)	*Ne* (CI)	*Ne*_cor_
**Española** (30) *Pcrit* = 0.03	**342.9** (334.4–351.9)	**358**
**Floreana** (30) *Pcrit* = 0.03	**1641** (1464.1–1866.2)	**1711**
**West** (107) *Pcrit* = 0.005	**7415.3** (6853.5–8077.1)	**7732**

*Pcrit* indicates the optimal minor allele frequency threshold based on sample size used to estimate *Ne*.

## Discussion

The Galapagos archipelago and the Galapagos bullhead shark were used as a model system to provide novel insight into the evolutionary processes that shape genetic structure and biogeographic patterns of shallow-water marine organisms in oceanic islands. Sequential island formation gradually established contemporary depth barriers between islands that varied in strength due to historical sea level fluctuations. This resulted in at least three distinct genetic clusters that exhibit low genetic diversity and effective population sizes that decrease from larger to smaller islands and with stronger isolation through historical and contemporary seascape configuration.

### Isolation by depth

The application of IBR analysis to a marine model system showcases the impact of contemporary and historical ocean bathymetry on genetic connectivity in coastal marine species with limited dispersal. Galapagos bullhead sharks showed contemporary genetic connectivity along short distances (20 km) of continuous coastlines and across less than 10 km of 100 m deep water, between Isabela and Fernandina Islands, but larger ocean depths between islands pose effective barriers at distances of only 50 km. Recently, IBR analyses were applied to identify ocean depth as barrier to gene flow at hundreds of kilometers in a reef-associated pelagic shark (*Carcharhinus amblyrhynchos)* while suitable shallow-water habitats acted as connectivity corridors (Boussarie et al. 2022). Similar to our study species, other benthic sharks (*Squatina californica*) and rays (*Utobatis halleri*) with low dispersal capacity and shallow depth distributions can maintain connectivity along continuous coastlines but show genetic differences across deep water between islands and islands and continental coasts at less than 50 km distance (Gaida 1997; Plank et al. 2010). Deeper water separating individual islands at a distances between 30 and 80 km and a minimum depth of 500 m, and islands from mainland locations at a distance of less than 20 km and a maximum depth of 200 m has also been found to generate genetic structure in a shallow-water bony fish without planktonic larval stage (Bernardi 2000). Further, genetic differences between individual islands of archipelagos has been reported at less than 100 km in marine invertebrates (Macaronesian archipelagos in the North Atlantic) and endemic intertidal bony fish (Galápagos) with limited dispersal (Bernardi et al. 2014; Vieira et al. 2019). However, no genetic structure was found between California horn sharks (*Heterodontus francisci*) sampled from different islands within the Channel Island archipelago separated by up to 100 km and 800 m deep water, despite showing strong genetic differences between the coast of California and the archipelago across 200 m deep water and at a distance of less than 20 km, unprecedented for any elasmobranch species (Canfield et al. 2022). Benthic Port Jackson sharks (*Heterodontus portusjacksoni*) migrate up to 1000 km along the Australian coast and manage to cross the Bass Strait to Tasmania, likely because the strait is relatively shallow (on average 60 m) and has several islands that can be used as stepping stones (Bass et al. 2016). Our study supports previous findings that ocean depth may limit dispersal in shallow-water elasmobranchs at short geographic distances, while oceanic and deep sea species have been found to maintain connectivity across depth barriers (Gubili et al. 2016; Domingues et al. 2018; Hirschfeld et al. 2021). Ocean depth plays a minor role in shaping connectivity of shallow-water marine organisms with juvenile larvae that use ocean currents to sustain dispersal between shallow-water habitat across various levels of bathymetry (Galarza et al. 2009). In contrast, for species that lack juvenile larvae, such as coastal sharks and rays, bathymetry can play a similar role in marine connectivity as topography does in some terrestrial systems. For instance, terrestrial sky islands are high altitude habitats that are separated by lower elevation. Genetic isolation among sky islands is common in a range of taxa, including insects (Smith and Farrell 2005) reptiles (Holycross and Douglas 2007) and amphibians (Osborne et al. 2019) and can be attributed to low dispersal capacity and a limited tolerance to environmental conditions in lower elevations (Polato et al. 2018). In sky islands, climatic fluctuations have altered connectivity through elevational shifts in environmental conditions, similar to the effect of sea level oscillations in oceanic islands (Rijsdijk et al. 2014; Mastretta-Yanes et al. 2015). In oceanic islands, shallow-water marine species with limited dispersal are likely to produce genetic patterns that are more similar to some terrestrial organisms compared to marine organisms with larvae that disperse with ocean currents.

### Flickering connectivity in oceanic archipelagos

The periodical fusion and fission of landmasses through historical sea-level fluctuations may have played a major role in shaping coastal marine populations in oceanic islands. In the Galapagos, low sea levels during the Pleistocene (2,588,000 to 11,700 years ago) repeatedly connected landmasses of the central and western archipelago and exposed seamounts between the central and southeastern islands (Parent et al. 2008; Geist et al. 2014). The paleogeographic IBR analysis showed that historical sea level fluctuations likely created dispersal corridors between the western and central islands and sea mounts provided stepping stones for the dispersal of Galapagos bullhead sharks between the central and southeastern islands. Similar admixture proportions among individual sharks within each genetic cluster imply that dispersal occurred before the establishment of a currently impermeable barrier and sufficient time has passed to homogenize genetic material received from other regions. In comparison, recent dispersal would result in distinctive admixture patterns. For example, recent dispersal events between islands resulted in two genetic clusters on the same island in Galapagos giant tortoises and marine iguanas, and hybridization among subspecies in the latter (Steinfartz et al. 2009; Poulakakis et al. 2012; Macleod et al. 2015). Dispersal and connectivity are subject to climatic variations in both marine and terrestrial systems. In terrestrial systems, climatic fluctuations caused the repeated fusion and fission of high altitude environments that shift to lower elevations during glacial cycles (Hazzi et al. 2018). This mechanism generated recurring population connectivity in mountain salamanders in the sky islands of New Mexico (Osborne et al. 2019) and rodents in East African mountain ranges (Bryja et al. 2014). It also created the flickering connectivity system of the Andean alpine biome called *Páramo* and is largely responsible for its extraordinary diversity (Flantua et al. 2019). Historical fluctuations in global climate also altered island configuration with the rise and fall of sea levels and left its imprint on genetic divergence and biogeography of Galapagos’ terrestrial fauna (Ali and Aitchison 2014). This process may also drive patterns in marine organisms that inhabit oceanic islands around the globe, but there was no clear empirical evidence prior to this study (Weigelt et al. 2016; Vieira et al. 2019). The case of Galapagos bullhead sharks exemplifies that flickering connectivity systems of oceanic archipelagoes not only shape the evolution of terrestrial species but also coastal marine organisms with limited dispersal.

### Island formation and genetic drift

Genetic population structure in Galapagos bullhead sharks likely reflects the gradual separation of individual islands and sequential vicariance events rather than progressive dispersal and colonization of newly formed islands. Paleogeographic reconstructions of the Galapagos archipelago that account for historical climate, noting the uncertainty of the models due to the lack of geologic data available, suggest a sequential separation of individual islands from a central island cluster (Karnauskas et al. 2017). Galapagos bullhead sharks may have colonized the central island cluster prior to the separation of the oldest island, Española, between 2 and 1.5 million years ago. Subsequently, individual islands separated sequentially, gradually forming bathymetric barriers that slowly reduced migration rates and increased genetic drift, resulting in four distinct genetic clusters. Historical reconnections during lower sea levels, and potentially the lower sample size for San Cristóbal Island, marginally altered the general pattern. Bayesian admixture analyses first differentiated Española, then Floreana, and then San Cristóbal Island from the western archipelago (Fig. 3 and Supplementary Information Figs. S4 and S5). However, in contrast to Floreana Island and the western archipelago, San Cristobal Island consistently showed greater admixture with the oldest Island, Española. But it also showed genetic admixture with Floreana Island and the western archipelago. Admixture results and a stronger differentiation based on genetic differentiation index (*D*_ST_) between San Cristóbal, compared to Floreana Island, and the western archipelago, indicate that San Cristóbal Island may have separated second in sequence but partially reconnected to Floreana and the western archipelago during lower sea levels. To reinforce the sequential vicariance viewpoint we consider four alternative scenarios that have shaped genetic patterns in Galapagos fauna: (1) High levels of inter-island dispersal, (2) recent arrival, (3) multiple colonization events, and (4) strong divergent selection (Juan et al. 2000; Parent et al. 2008).

In the first scenario, high dispersal rates found in mobile marine species, for example Galapagos Penguins (Akst et al. 2002) and Galapagos sea lions (Wolf et al. 2008), lead to high genetic connectivity between islands that are separated by deep ocean. In contrast, species with limited capacity to swim over open ocean, may show a sequential divergence from older to younger islands (Parent et al. 2008; Poulakakis et al. 2020). This general pattern can be interrupted by occasional inter-island migrations, which was apparent in Galapagos marine iguanas but not Galapagos bullhead sharks (Steinfartz et al. 2009; Macleod et al. 2015).

Recent colonization and subsequent expansion throughout the archipelago, the second alternative, resulted in inter-island differentiation over the last 125,000 years in Galapagos hawks, a bird with limited over-water dispersal (Bollmer et al. 2005). Although, this scenario could generate the isolation by depth pattern in Galapagos bullhead sharks, it would also result in population bottlenecks and directional gene flow towards islands that are colonized after the arrival of a small founder population, which was not apparent in this study (Clegg et al. 2002; Chaves et al. 2012). However, detecting recent or mild bottlenecks is challenging in populations with recent range expansion in non-model organisms with structured metapopulations and estimating directional gene flow in non-equilibrium populations is limited using the *divMigrate* approach (Sundqvist et al. 2016; Maisano Delser et al. 2019; Lesturgie et al. 2023). Future application of demographic history modeling may be useful to further examine this scenario for Galapagos bullhead sharks.

The third alternative is the occurrence of multiple colonization events from continental ranges to oceanic islands, which commonly result in the genetic divergence among paraphyletic groups that reflect a sequential island formation pattern (Emerson 2002; Schluter 2009; Schluter and Conte 2009). Paraphyletic groups have been found in Galapagos lava lizards and leaf-toed geckos (Benavides et al. 2009; Torres-Carvajal et al. 2014). However, samples from the continental coast of South America will be required to rule out multiple colonization events in Galapagos bullhead sharks in the future (Emerson 2002).

Genetic diversification through natural selection and adaptation to the environment, the fourth alternative, is common in terrestrial, but less common in marine organisms that colonize oceanic archipelagos (Pinheiro et al. 2017; Hedrick 2019). Outlier SNPs may represent genetic variants that are putatively selected for by the environment (Nielsen et al. 2009; Allendorf et al. 2010) and showed stronger spatial genetic structure compared to neutral SNPs at the scale of ocean basins in pelagic teleosts and sharks (Pazmiño et al. 2018; Pecoraro et al. 2018). Within the Galapagos archipelago, stronger genetic structure was discovered using outlier compared to neutral loci in an endemic shallow-water bony fish, suggesting adaptation to local environments of individual islands (Bernardi 2022). The discovery of genomic regions putatively under selection in non-model organisms was limited by the sampling, sequencing and statistical methods applied here (Tiffin and Ross-Ibarra 2014; Lowry et al. 2017; Ahrens et al. 2018). But the diverse oceanographic conditions of the Galapagos’ bioregions (see Fig. 1) may provide a suitable scenario for future research to examine adaptive genetic variation in elasmobranchs with low to intermediate dispersal ability and inter-island connectivity, and low rates of immigration to the oceanic archipelago (Edgar et al. 2004; Pinheiro et al. 2017).

In conclusion, the possible lack of recent bottlenecks and directional gene flow, and the absence of paraphyletic groups underpin that single colonization of a central island cluster and gradual genetic drift among sequentially separating islands, influenced by historical sea level changes, is the most likely scenario for genetic divergence in Galapagos bullhead sharks. As more precise paleogeographic models become available demographic history and coalescent analyses could be applied to examine the sequence and timing of divergence in Galapagos bullhead sharks and validate the patterns presented here.

### Genomic signatures of isolation

Genomic signatures of isolation in Galapagos bullhead sharks are consistent with the gradual formation of barriers to dispersal and resemble those typical of terrestrial island biogeography. In oceanic islands, species with low dispersal commonly have lower genetic diversity and smaller population sizes compared to mainland populations owing to the reduced genetic variation of few founding individuals and because limited resources in small and fragmented habitats sustain smaller populations (Frankham 1996, 1997). Galapagos bullhead sharks show lower diversity, based on similar sequencing techniques and numbers of markers, compared to shark species with higher dispersal or that were sampled along continental ranges (Supplementary Information Table S5). However, comparing levels of genomic diversity, based on SNP markers, among studies remains a challenge (Cariou et al. 2016; Schmidt et al. 2021). Estimating genome-wide heterozygosity (Schmidt et al. 2021) in elasmobranchs from publicly available data sets and future island-mainland comparisons in our study species may confirm lower genomic diversity in marine island populations. In some cases relatively high genetic diversity in island populations is also possible. For example, large numbers of founding individuals in island-colonizing song birds, or high dispersal rates and a large population size that offset the founder effect in Christmas Island red crabs (*Gecarcoidea natalis*), led to relatively high genetic diversity (Aleixandre et al. 2013; Weeks et al. 2014). Galapagos bullhead sharks have low dispersal capacity and smaller populations sizes, in comparison. Therefore, low genomic diversity may be traced back to a founder effect caused by few colonizing individuals, a common feature in terrestrial island populations (Frankham 1997). Considering its poor dispersal ability, Galapagos bullhead sharks or their egg cases attached to debris could have been transported from the South American coast to the Galapagos on rafts drifting with the Humboldt Current, similar to the mechanism proposed for many native terrestrial vertebrates (Ali and Fritz 2021). Another parallel between Galapagos bullhead sharks and terrestrial species are effective populations sizes (*N*_*e*_) that scale with habitat availability in oceanic archipelagos (Frankham 1997; Brüniche-Olsen et al. 2019). In some sharks, *N*_*e*_ approximates the census size (*N*_*c*_) because of their low fecundity, consistent reproductive success, long life spans and late age at maturity (Portnoy et al. 2009; Waples et al. 2013; Dudgeon and Ovenden 2015), a life history also characteristic of Heterodont sharks (McLaughlin and O’Gower 1971; Powter and Gladstone 2008). This contrasts with many marine species because they have high dispersal rates, large-scale genetic connectivity, high fecundity, and large population sizes (Palumbi 1994). Large populations of marine species have genetic effective population sizes (*N*_*e*_) that are orders of magnitude smaller than the true number of adults in the population (*N*_*c*_) owing to the high fecundity and variability in reproductive success (Palstra and Ruzzante 2008; Waples et al. 2016). Comparing *N*_*e*_ estimates among elasmobranch species is challenging due to the effect of different life histories, migration rates, sampling design, and sequencing techniques on estimates (Waples et al. 2013; Dudgeon and Ovenden 2015; Marandel et al. 2019). For example, pelagic blue sharks in the Atlantic have a high *N*_*c*_ but showed *N*_*e*_ estimates around 5000, lower than our estimates for the western archipelago (King et al. 2015; Veríssimo et al. 2017). Genetic effective population size estimates for Sandbar sharks were only slightly lower, between 1500 and 4900 and similar to *N*_*c*_ (Portnoy et al. 2009). This may be explained by higher migration rates, large litter size, earlier age at maturity, and longer adult life spans that result in a lower N_e_/N_c_ ratio in blue compared to sandbar sharks (Portnoy et al. 2009, 2010; Taguchi et al. 2015). When compared to another benthic elasmobranch, Galapagos bullhead sharks had smaller *N*_*e*_ estimates than blue skates sampled at inshore locations (21,000), but similar estimates (3500) than populations at some isolated offshore locations (Delaval et al. 2022). While our results suggest that Galápagos bullhead sharks may lack adaptive potential at the most isolated locations, future comparisons of *N*_*e*_ between the Galapagos and the continental coast and between *N*_*e*_ and *N*_*c*_ are needed to corroborate these results (Ryman et al. 2019). The life history of Galapagos bullhead sharks, and lack of dispersal and genetic connectivity therefore suggest that *N*_*e*_ estimates for individual islands approximate census sizes. Low genetic diversity and population sizes that scale to the amount of available resources are common in terrestrial island biogeography but, to our knowledge, unprecedented in marine organisms (Dawson 2016).

## Conclusion

This study shows that Galapagos bullhead sharks produce genetic and biogeographic signatures comparable to many terrestrial organisms in oceanic archipelagos and contrasting those commonly found in pelagic elasmobranchs and marine taxa with larval dispersal. Isolated marine populations with small effective population sizes and low genetic diversity are at high risk of extinction because they have reduced adaptive potential and lack replenishment from adjacent locations (Frankham et al. 2014; Ryman et al. 2019). This highlights the importance of preserving shallow marine habitat to protect marine island populations (Vieira et al. 2019). Future research on marine species with similar characteristics to our study species may broaden our understanding of island evolution and biogeography in the marine realm and enhance efforts to preserve the biodiversity of oceanic archipelagos.

### Data archiving

The data used in this study, including genotyped and filtered SNP data, and details of data analyses are openly available through the Supplementary Information and datadryad.org 10.5061/dryad.dv41ns241.

## Supplementary information

SUPPLEMENTAL MATERIAL
