# Supplementary material for: What Darwin could not see: island formation and historical sea levels shape genetic divergence and island biogeography in a coastal marine species
Source: Heredity (Edinb). 2023 Jul 3;131(3):189–200. doi: 10.1038/s41437-023-00635-4 (PMC10462691; doi:10.1038/s41437-023-00635-4)
Supplement: Supplementary file 1 — SUPPLEMENTAL MATERIAL [file 41437_2023_635_MOESM1_ESM.docx]

**Supplementary Information for:**

**What Darwin couldn’t see: Island formation and historical sea levels shape genetic divergence and island biogeography in a coastal marine species**

Maximilian Hirschfeld^1,2*^, Adam Barnett^1,3,4^ , Marcus Sheaves^1, 3^, Christine Dudgeon^4,5^

^1^College of Science and Engineering, James Cook University, Townsville, Queensland, Australia

^2^Galápagos Science Center, Universidad San Francisco de Quito, Isla San Cristóbal, Galápagos, Ecuador

^3^Marine Data Technology Hub, James Cook University, Townsville, Queensland, Australia

^4^Biopixel Oceans Foundation, Cairns Queensland, Australia

^5^The University of Queensland, School of Biomedical Sciences, Saint Lucia, Queensland, Australia

*Corresponding author

**Table of Contents:**

| **FIGURE S1. Map of dive surveys** | Page 2 |
| --- | --- |
| **SNP genotyping and quality control** | Page 3-4 |
| **Isolation by depth - Resistance surfaces** | Page 5-7 |
| **TABLE S3. Pairwise comparisons (*F_ST_* and *D_ST_*)** | Page 8 |
| **FIGURE S2. Admixture neutral SNPs full data set** | Page 9 |
| **FIGURE S3. Admixture neutral SNPs reduced data set** | Page 10 |
| **TABLE S4. Genomic diversity of sharks** | Page 11 |
| **References** | Page 12-13 |

**FIGURE S1.** All 33 dive locations surveyed between 2015 and 2018.


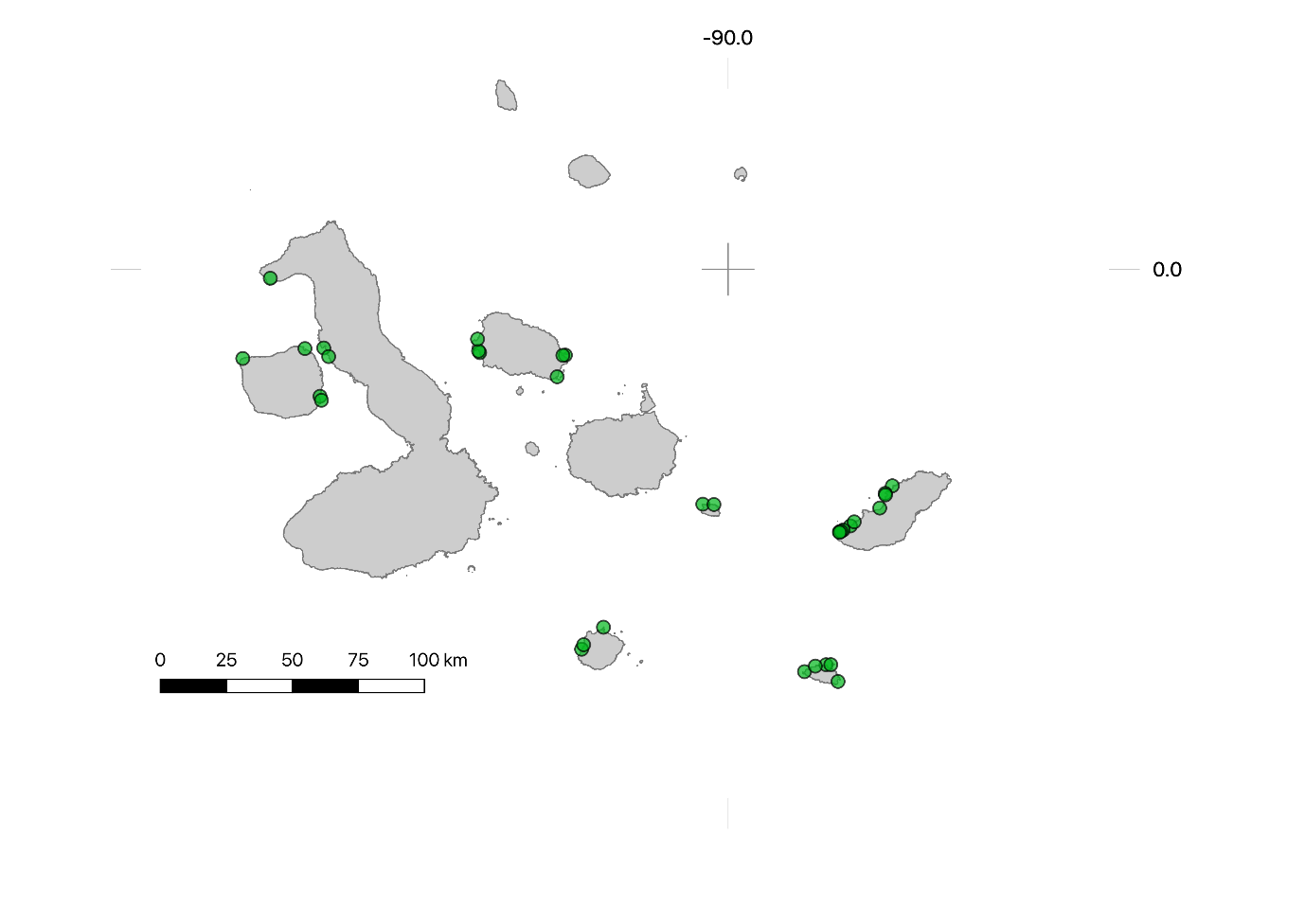


**SNP genotyping and quality control (see TABLE S1)**

Single nucleotide polymorphisms (SNPs) were genotyped with the proprietary Diversity Array Technologies Pty Ltd (DArT, Canberra, Australia) analytical pipelines [1–3]. Additionally, we randomized tissue samples from all sampling locations across sequencing plates and replicated tissue samples from two individuals within and across sequencing plates to independently assure genotyping consistency and generate baseline values to quickly assess relatedness and potential sample contamination during data filtering [4–7]. The raw data set was filtered using the R package *radiator* [8]. First, SNPs below 98% reproducibility and markers that were not present in all sampling locations were removed. We excluded markers with a low minor allele count (MAC<4) to reduce genotyping error while retaining a higher probability of discovering outlier loci putatively under selection compared to applying a commonly used 5 % minor allele frequency (MAF) threshold [9]. Further, SNPs with a coverage (read depth) below 10 and above 50, and a call rate of less than 95% were removed. We reduced the likelihood of physical linkage by keeping only one SNP per sequence. Departures from Hardy-Weinberg-Equilibrium (HWE) were tested using the *HardyWeinberg* R package [10–12]. Markers were removed that were out of HWE in at least three sampling locations based on a mid p-value threshold of 0.05. Moreover, we identified and removed putatively sex-linked SNP markers with the *sexy_markers* function in the *radiator* R package [8]. To identify makers that are putatively under selection (outlier SNPs) we first applied the individual based method of the *PCadapt* R package [13]. A minor allele frequency threshold of 5% was used to derive p-values. Outliers were selected that fell below a 5% percent false discovery rate (FDR) based on q-values, which are transformed from p-values, using the R package *qvalue* [14]. We further used OutFLANK, which constructs a null distribution of loci that generate population structure through neutral processes and then iteratively trims loci that are putatively under selection, resulting in fewer false positives compared to *F*_ST_ based methods [15]. We ran OutFLANK using the *gl.outflank* wrapper function in the R package *DartR* [16]. To avoid bias we excluded samples from locations with small sample size (Cabo Douglas and Santiago) prior to the analysis. We trimmed the upper and lower 5% of loci and excluded loci with less than 10% expected heterozygosity to generate the null distribution and applied a 5% FDR threshold [15]. Loci that were identified as putative outliers with both methods were then removed to create a neutral SNP data set to assess genetic patterns generated through neutral processes. Two outlier SNP data sets were used to explore the role of selection. One set contained outlier SNPs identified by both outlier methods, which is less likely to contain false positives, but may be too conservative to retain sufficient markers to detect genetic structure caused by divergent selection. To assure results were not biased by the conservative outlier detection approach, we created another, less conservative data set, which contained outlier SNPs detected by PCadapt only.

**TABLE S1.** SNP filtering steps, corresponding thresholds, and resulting number of SNPs and individuals kept in the data set. Tow data sets, outlier SNPs detected by both outlier methods and neutral SNPs excluding the outliers, were retained for analyses.

| **Filter** | **Values/thresholds** | **SNPs (samples) retained** |
| --- | --- | --- |
| Raw data |  | 33606 (188) |
| DArT reproducibility | 98% | 31196 (188) |
| Common markers |  | 29522 (188) |
| MAC | MAC=4 | 12423 (188) |
| Coverage | Min 10 max 50 | 10396 (188) |
| Genotyping (call rate) | 0.05 | 9742 (188) |
| SNPs thinning (short linkage) | keep one SNP with lowest MAC | 9280 (188) |
| Heterozygosity | None removed | 9280 (188) |
| detect duplicate genomes | Duplicate samples (n=6)  Recaptures (n=2) | 9280 (180) |
| Filter HWE | In 3 populations/0.05 mid p-value | 9239 (180) |
| Sex-linked markers | 5 | 9234 (180) |
| **Total number of SNPs and individuals retained** |  |  |
| **Outlier data set** |  | **11** (180) |
| **Neutral data set** |  | **9223** (180) |

**Isolation by depth**

To test for the effect of contemporary bathymetry and historical sea level fluctuations on genetic connectivity we adapted isolation by resistance (IBR) analysis [17] using depth profiles of the Galapagos to represent landscape resistance to animal dispersal. Briefly, two IBR models were built using single surface optimization in the ResistaceGA R package, one based on contemporary bathymetry and another based on paleogeographic bathymetry that accounts for historical sea level fluctuations. We compared the resistance models to a null model based on isolation by distance analyses that only uses geographic distance and does not account for potential depth barriers [18–20]. Geographic distance was measured as straightest over-water distance between sampling locations using simple least cost analysis in the R package *marmap* [21]. Next we used a high resolution (15 arc-seconds) digital elevation model (DEM) of the Galapagos archipelago obtained from GEBCO (General Bathymetric Chart of the Oceans) to create the contemporary and paleogeographic isolation by resistance models [22]. The DEM was reclassified using the *raster* R package [23]. Because of the lack of accurate representation of shallow coastal areas in the data set we assigned a shallow water depth of 1m to an area of a single pixel extent along the coastline of the islands. All remaining land surface was assigned “NA”, representing a complete barrier to dispersal. Paleogeographic models of the Galapagos archipelago spanning the last 700 thousand years estimated extreme sea level low stands were repeatedly between 145 and 210 m deeper during glacial maxima [24]. To test if low sea levels during glacial periods may have facilitated historical dispersal, the paleogeographic IBR model was built by optimizing a bathymetry layer where all areas between 0 and -210 m, corresponding to areas with shallow water depth at least one time during the last 700 thousand years, were assigned a shallow water depth of 1m. Resistance distances (Shah and McRae, 2008) were calculated for optimized contemporary and paleogeographic resistance surfaces with the *ResistanceGA* package (Peterman, 2018). *ResistanceGA* uses a genetic algorithm to optimize resistance surfaces based on pairwise genetic data and resistance distances generated through CIRCUITSCAPE software (Peterman, 2018)(Anantharaman *et al.*, 2019). The linear relationship between linearized genetic distances (FST/(1 FST) and straightest over-water distances and between linearized genetic distances and contemporary and paleogeographic resistance distances was plotted and quantified using Pearson’s correlation coefficient (*r^2^*) and Mantel tests with 1000 permutations with the R package *DartR* [16]. The performance of each model was compared using a causal modeling approach [27,28].

**FIGURE S2:**

a) Reverse monomolecular data transformation identified as transformation that best explained genetic differences using ResitanceGA single surface optimization of the contemporary bathymetry of the Galapagos.

**
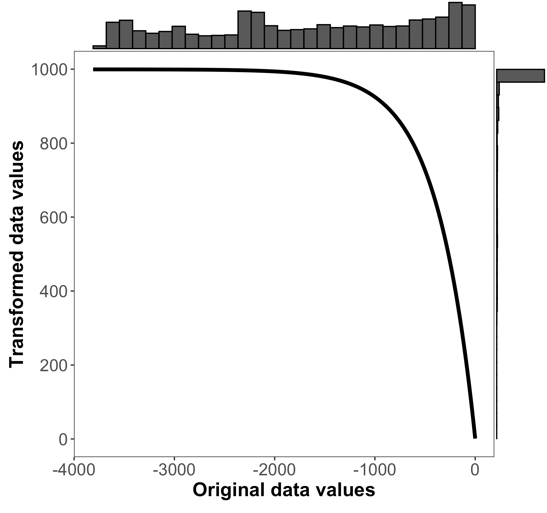
**

b) Diagnostic plot of model fit for genetic distance (linearized FST) versus contemporary resistance distance based on resistance surface optimized with reverse monomolecular data transformation.

**
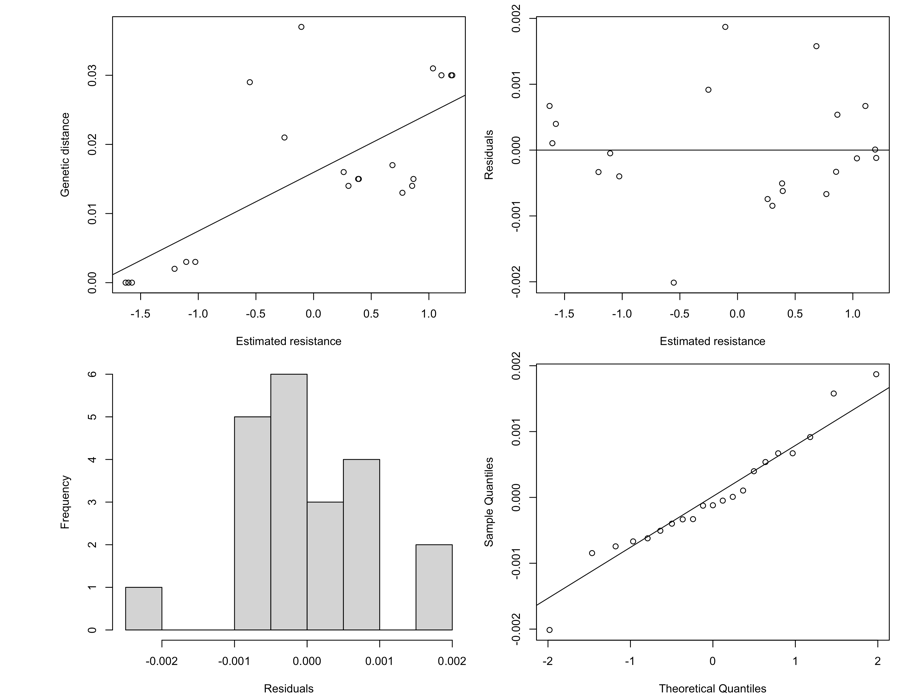
**

**TABLE S2:** Fit statistics of the contemporary IBR model based on transformed (reverse monomolecular) contemporary bathymetry surface.

| **Surface** | **k** | **AICc** | **R2m** | **R2c** | **LL** |
| --- | --- | --- | --- | --- | --- |
| Transformed contemporary bathymetry | 4 | -161.59031 | 0.15987558 | 0.98343329 | 94.7951549 |

**Abbreviations:** Number of model parameters (k), Akaike’s information criterion adjusted for small-sample sizes (AICc), marginal R2 (R2m), conditional R2 (R2c), loglikelihood (LL).

**FIGURE S3:**

a) Reverse monomolecular data transformation identified as transformation that best explained genetic differences using ResitanceGA single surface optimization of the paleogeographic bathymetry of the Galapagos.

**
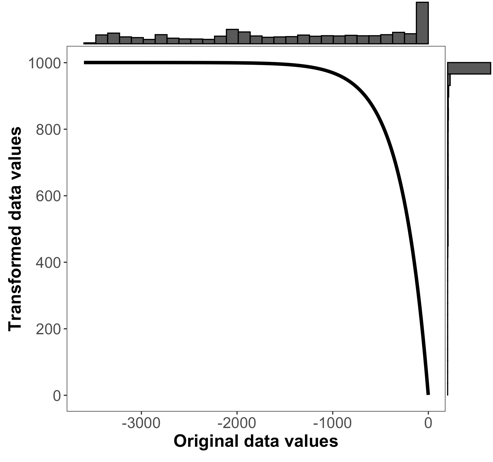
**

b) Diagnostic plot of model fit for genetic distance (linearized FST) versus paleogeographic resistance distance based on resistance surface optimized with reverse monomolecular data transformation.

**
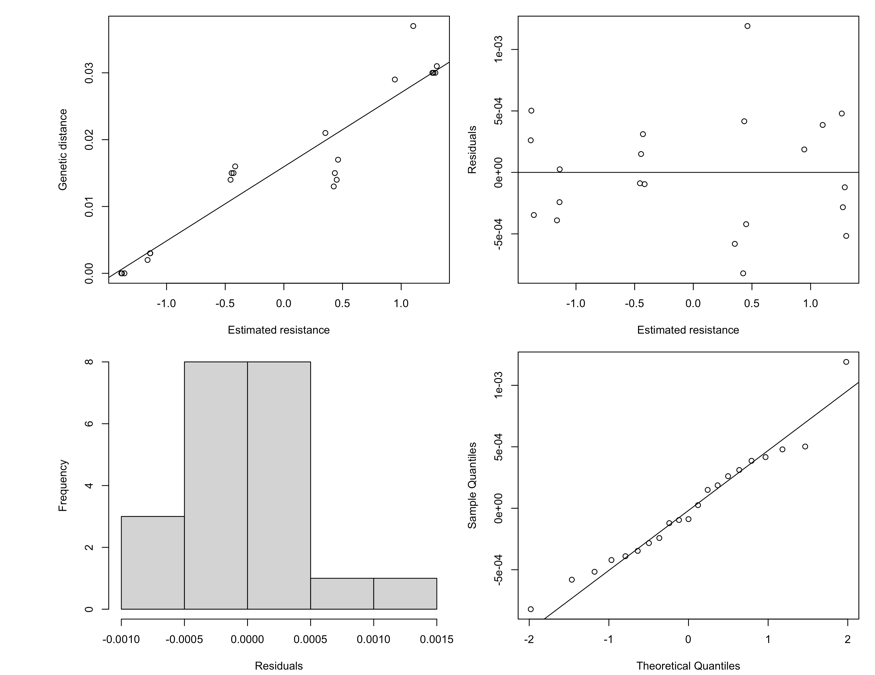
**

**TABLE S3:** Fit statistics of the paleogeographic IBR model based on transformed (reverse monomolecular) paleogeographic bathymetry surface.

| **Surface** | **k** | **AICc** | **R2m** | **R2c** | **LL** |
| --- | --- | --- | --- | --- | --- |
| Transformed paleogeographic bathymetry | 4 | -186.94732 | 0.7542839 | 0.99558125 | 107.473662 |

**Abbreviations:** Number of model parameters (k), Akaike’s information criterion adjusted for small-sample sizes (AICc), marginal R2 (R2m), conditional R2 (R2c), loglikelihood (LL).

**TABLE S4.** Pairwise genetic fixation index (*F*_ST_) index below, and corresponding p-values, above diagonal (top table). Pairwise genetic differentiation index (*D*_ST_) below, and corresponding p-values, above diagonal (bottom table). Bold numbers indicate significant values after Benjamin-Hochberg false discovery correction.

***F*_ST_**

|  | **ES** | **FL** | **PE** | **PM** | **PNG** | **PVR** | **SCY** |
| --- | --- | --- | --- | --- | --- | --- | --- |
| **ES** |  | 0.001 | 0.001 | 0.001 | 0.001 | 0.001 | 0.001 |
| **FL** | **0.0368** |  | 0.001 | 0.001 | 0.001 | 0.001 | 0.001 |
| **PE** | **0.0304** | **0.0149** |  | 0.27 | 0.84 | 0.001 | 0.001 |
| **PM** | **0.0303** | **0.0150** | 0.0004 |  | 0.29 | 0.001 | 0.001 |
| **PNG** | **0.0297** | **0.0139** | -0.0004 | 0.0003 |  | 0.001 | 0.001 |
| **PVR** | **0.0315** | **0.0161** | **0.0027** | **0.0028** | **0.0023** |  | 0.001 |
| **SCY** | **0.0289** | **0.0207** | **0.0140** | **0.0149** | **0.0133** | **0.0169** |  |

***D*_ST_**

|  | **ES** | **FL** | **PE** | **PM** | **PNG** | **PVR** | **SCY** |
| --- | --- | --- | --- | --- | --- | --- | --- |
| **ES** |  | 0.001 | 0.001 | 0.001 | 0.001 | 0.001 | 0.001 |
| **FL** | ***0.00079*** |  | 0.001 | 0.001 | 0.001 | 0.001 | 0.001 |
| **PE** | ***0.00061*** | ***0.00032*** |  | 0.78 | 0.27 | 0.001 | 0.001 |
| **PM** | ***0.00062*** | ***0.00032*** | 0.00009 |  | 0.24 | 0.001 | 0.001 |
| **PNG** | ***0.00064*** | ***0.00030*** | 0.0001 | 0.0001 |  | 0.001 | 0.001 |
| **PVR** | ***0.00063*** | ***0.00034*** | ***0.00014*** | ***0.00014*** | ***0.00012*** |  | 0.001 |
| **SCY** | ***0.00064*** | ***0.00057*** | ***0.00042*** | ***0.0004*** | ***0.00038*** | ***0.00047*** |  |

**FIGURE S4.** Top: The most likely number of *K* ancestral populations indicated by the lowest cross-entropy criterion generated for 180 sharks and 9223 neutral SNPs using the R package *tess3R*. Bottom: Admixture proportions of *K* 2-4 ancestral populations for 180 sharks and 9223 neutral SNPs.


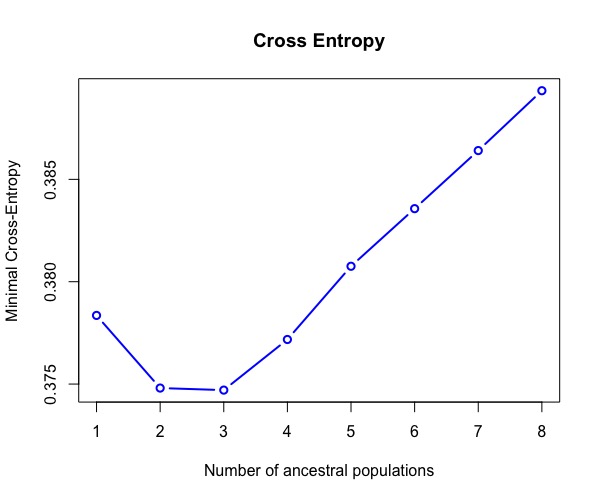


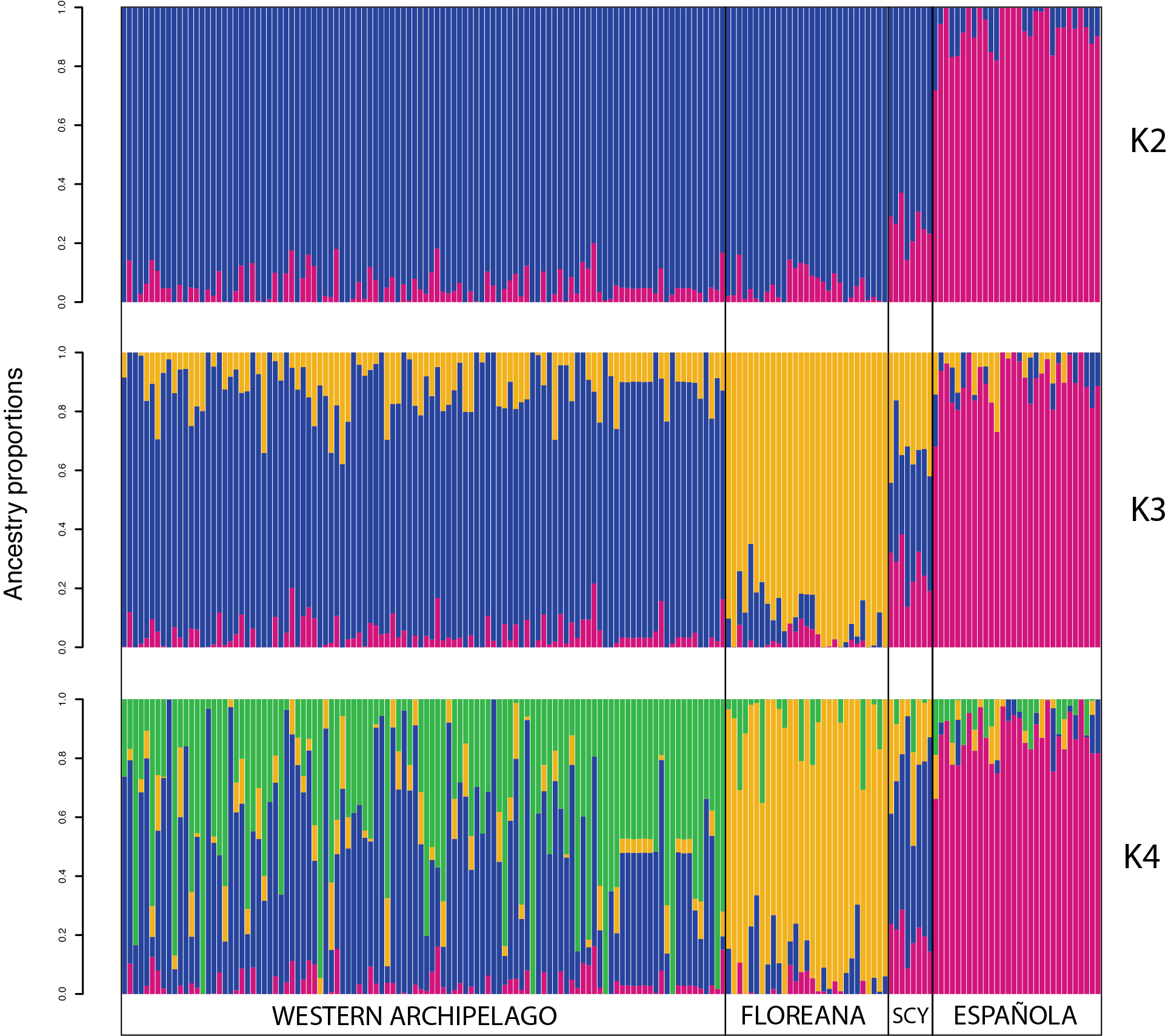


**FIGURE S5.** Top: The most likely number of *K* ancestral populations indicated by the lowest cross-entropy criterion generated for the subset of 56 sharks and 9223 neutral SNPs. Bottom: Admixture proportions of *K* 2-5 ancestral populations the subset of 56 sharks and 9223 neutral SNPs.


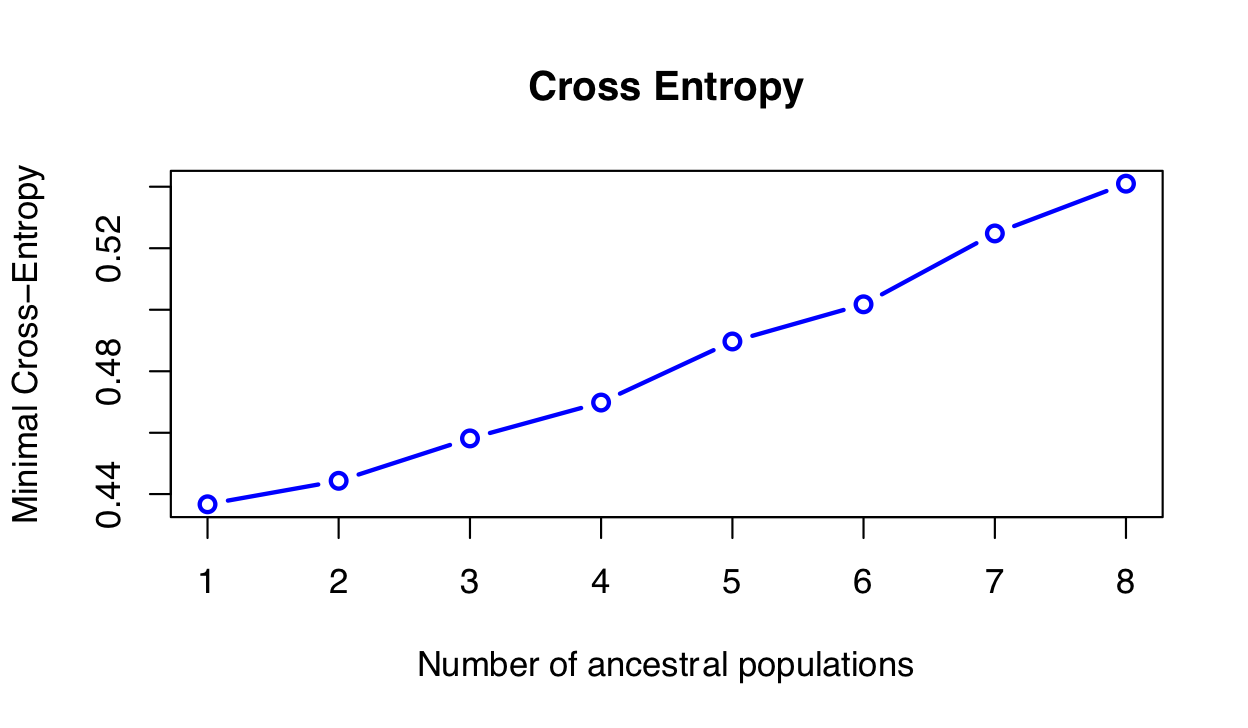


**
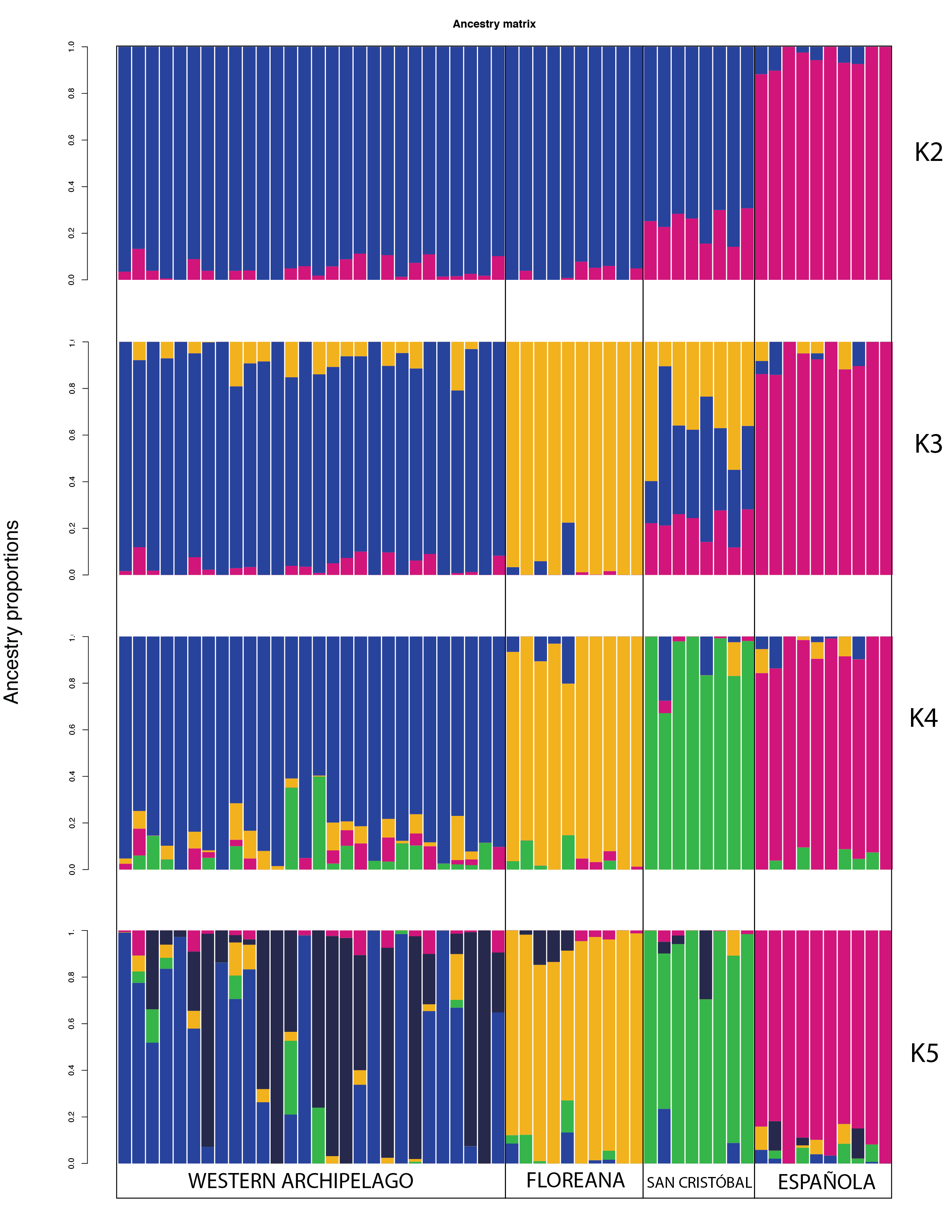
**

**TABLE S5.** Genomic diversity of shark species studied using similar genotyping approaches.

| **Species** | **Genotyping approach** | **SNPs/individuals** | **Ho (Range)** | **Reference** |
| --- | --- | --- | --- | --- |
| *Galeorhinus galeus* | DArTseq* | 6587/76 | 0.264- 0.265 | [29] |
| *Glyphis glyphis* | DArTseq | 1330/356 | 0.2597-0.2623 | [30] |
| *Carcharhinus albimarginatus* | ddRAD [31] | 6461/ 92 | 0.126 - 0.13 | [32] |
| *Carcharhinus brachyurus* | DArTseq | 3766/106 | 0.208 - 0.261 | [33] |
| *Carcharhinus obscurus* | DArTseq | 8886/207 | 0.159 - 0.200 | [33] |
| *Carcharhinus amblyrhynchos* | DArTseq | 4798/170 | 0.288 - 0.312  (0.139 in Chagos Archipelago) | [34] |
| *Carcharhinus galapagensis* | DArTseq | 8103/85 | 0.188 - 0.193 | [35] |
| *Carcharhinus galapagensis* | DArTseq | 7784/206 | 0.202- 0.237 | [36] |
| *Scyliorhinus canicula* | 2b-RAD [37] | 2674/71 | 0.53 - 0.73 | [38] |
| *Sphyrna lewini* | DArTseq | 5689/310 | 0.116 – 0.130 | [39] |
| *Heterodontus quoyi* | DArTseq | 9223/180 | 0.089-0.098 | This study |

* DartSeq methods and references can be found in the main manuscript.

**References:**

1. Sansaloni C, Petroli C, Jaccoud D, Carling J, Detering F, Grattapaglia D, Kilian A. 2011 Diversity Arrays Technology (DArT) and next-generation sequencing combined: genome-wide, high throughput, highly informative genotyping for molecular breeding of Eucalyptus. *BMC Proc.* **5**. (doi:10.1186/1753-6561-5-s7-p54)

2. Kilian A *et al.* 2012 Diversity arrays technology: A generic genome profiling technology on open platforms. In *Methods in Molecular Biology 888*, pp. 67–89. (doi:https://doi.org/10.1007/978‐1‐61779‐870‐2)

3. Georges A *et al.* 2018 Genomewide SNP markers breathe new life into phylogeography and species delimitation for the problematic short-necked turtles (Chelidae: Emydura) of eastern Australia. *Mol. Ecol.* **27**, 5195–5213. (doi:10.1111/mec.14925)

4. Linck E, Battey CJ. 2019 Minor allele frequency thresholds strongly affect population structure inference with genomic data sets. *Mol. Ecol. Resour.* **19**, 639–647. (doi:10.1111/1755-0998.12995)

5. O’Leary SJ, Puritz JB, Willis SC, Hollenbeck CM, Portnoy DS. 2018 These aren’t the loci you’e looking for: Principles of effective SNP filtering for molecular ecologists. *Mol. Ecol.* **27**, 3193–3206. (doi:10.1111/mec.14792)

6. Waples RS, Allendorf F. 2015 Testing for hardy-weinberg proportions: Have we lost the plot? *J. Hered.* **106**, 1–19. (doi:10.1093/jhered/esu062)

7. Meirmans PG. 2015 Seven common mistakes in population genetics and how to avoid them. *Mol. Ecol.* **24**, 3223–3231. (doi:10.1111/mec.13243)

8. Gosselin T. 2019 radiator: RADseq Data Exploration, Manipulation and Visualization using R. (doi:10.5281/zenodo.1475182)

9. Ahrens CW, Rymer PD, Stow A, Bragg J, Dillon S, Umbers KDL, Dudaniec RY. 2018 The search for loci under selection: trends, biases and progress. *Mol. Ecol.* **27**, 1342–1356. (doi:10.1111/mec.14549)

10. Graffelman J. 2019 HardyWeinberg: Statistical Tests and Graphics for Hardy-Weinberg Equilibrium.

11. Graffelman J. 2015 Exploring Diallelic Genetic Markers: The {HardyWeinberg} Package. *J. Stat. Softw.* **64**, 1–23.

12. Graffelman J, Morales-Camarena J. 2008 Graphical tests for Hardy-Weinberg Equilibrium based on the ternary plot. *Hum. Hered.* **65**, 77–84.

13. Luu K, Bazin E, Blum MGB. 2017 pcadapt: an R package to perform genome scans for selection based on principal component analysis. *Mol. Ecol. Resour.* **17**, 67–77. (doi:10.1111/1755-0998.12592)

14. Storey JD, Bass AJ, Dabney A, Robinson D. 2019 qvalue: Q-value estimation for false discovery rate control.

15. Whitlock MC, Lotterhos KE. 2015 Reliable detection of loci responsible for local adaptation: Inference of a null model through trimming the distribution of FST. *Am. Nat.* **186**, S24–S36. (doi:10.1086/682949)

16. Gruber B, Georges A. 2019 dartR: Importing and Analysing SNP and Silicodart Data Generated by Genome-Wide Restriction Fragment Analysis.

17. McRae BH. 2006 Isolation By Resistance. *Evolution (N. Y).* **60**, 1551. (doi:10.1554/05-321.1)

18. Wright S. 1943 Isolation by Distance. *Genetics* **28**, 114–38.

19. Slatkin M. 1993 Isolation by Distance in Equilibrium and Non-Equilibrium Populations. *Evolution (N. Y).* (doi:10.2307/2410134)

20. Guillot G, Leblois R, Coulon A, Frantz AC. 2009 Statistical methods in spatial genetics. *Mol. Ecol.* **18**, 4734–4756. (doi:10.1111/j.1365-294X.2009.04410.x)

21. Pante E, Simon-Bouhet B. 2013 marmap: A Package for Importing, Plotting and Analyzing Bathymetric and Topographic Data in R. *PLoS One* **8**, 6–9. (doi:10.1371/journal.pone.0073051)

22. GEBCO Bathymetric Compilation Group. 2019 The GEBCO_2019 Grid - a continuous terrain model of the global oceans and land. (doi:10.5285/836f016a-33be-6ddc-e053-6c86abc0788e)

23. Hijmans RJ. 2019 raster: Geographic Data Analysis and Modeling.

24. Ali JR, Aitchison JC. 2014 Exploring the combined role of eustasy and oceanic island thermal subsidence in shaping biodiversity on the Galápagos. *J. Biogeogr.* **41**, 1227–1241. (doi:10.1111/jbi.12313)

25. Shah VB, McRae BH. 2008 Circuitscape : A Tool for Landscape Ecology. *Proc. 7th Python Sci. Conf.* (doi:10.1111/j.1523-1739.2008.00942.x)

26. van Etten J. 2017 R package gdistance: Distances and routes on geographical grids. *J. Stat. Softw.* **76**. (doi:10.18637/jss.v076.i13)

27. Cushman SA, McKelvey KS, Hayden J, Schwartz MK. 2006 Gene Flow in Complex Landscapes: Testing Multiple Hypotheses with Causal Modeling. *Am. Nat.* **168**, 486–499. (doi:10.1086/506976)

28. McRae BH, Beier P. 2007 Circuit theory predicts gene flow in plant and animal populations. *Proc. Natl. Acad. Sci. U. S. A.* **104**, 19885–19890. (doi:10.1073/pnas.0706568104)

29. Devloo-Delva F, Maes GE, Hernández SI, Mcallister JD, Gunasekera RM, Grewe PM, Thomson RB, Feutry P. 2019 Accounting for kin sampling reveals genetic connectivity in Tasmanian and New Zealand school sharks, Galeorhinus galeus. *Ecol. Evol.* **9**, 4465–4472. (doi:10.1002/ece3.5012)

30. Feutry P *et al.* 2017 Inferring contemporary and historical genetic connectivity from juveniles. *Mol. Ecol.* **26**, 444–456. (doi:10.1111/mec.13929)

31. Peterson BK, Weber JN, Kay EH, Fisher HS, Hoekstra HE. 2012 Double digest RADseq: An inexpensive method for de novo SNP discovery and genotyping in model and non-model species. *PLoS One* **7**. (doi:10.1371/journal.pone.0037135)

32. Green ME *et al.* 2018 Mixed-marker approach suggests maternal philopatry and sex-biased behaviours of narrow sawfish Anoxypristis cuspidata. *Endanger. Species Res.* **37**, 45–54. (doi:10.3354/esr00912)

33. Junge C *et al.* 2019 Comparative population genomics confirms little population structure in two commercially targeted carcharhinid sharks. *Mar. Biol.* **166**. (doi:10.1007/s00227-018-3454-4)

34. Momigliano P, Harcourt R, Robbins WD, Jaiteh V, Mahardika GN, Sembiring A, Stow A. 2017 Genetic structure and signatures of selection in grey reef sharks (Carcharhinus amblyrhynchos). *Heredity (Edinb).* , 1–12. (doi:10.1038/hdy.2017.21)

35. Pazmiño DA, Maes GE, Simpfendorfer CA, Salinas-de-León P, van Herwerden L. 2017 Genome-wide SNPs reveal low effective population size within confined management units of the highly vagile Galapagos shark (Carcharhinus galapagensis). *Conserv. Genet.* **18**, 1151–1163. (doi:10.1007/s10592-017-0967-1)

36. Pazmiño DA *et al.* 2018 Strong trans-Pacific break and local conservation units in the Galapagos shark (Carcharhinus galapagensis) revealed by genome-wide cytonuclear markers. *Heredity (Edinb).* **120**, 407–421. (doi:10.1038/s41437-017-0025-2)

37. Wang S, Meyer E, McKay JK, Matz M V. 2012 2b-RAD: a simple and flexible method for genome-wide genotyping. *Nat. Methods* **9**, 808–810. (doi:10.1038/nmeth.2023)

38. Manuzzi A, Zane L, Muñoz-Merida A, Griffiths AM, Veríssimo A. 2019 Population genomics and phylogeography of a benthic coastal shark (Scyliorhinus canicula) using 2b-RAD single nucleotide polymorphisms. *Biol. J. Linn. Soc.* **126**, 289–303. (doi:10.1093/biolinnean/bly185)
